# 2531 Phase II award: Evaluation of outcomes in preparing independent researchers by continued mentoring and career development support (2006–2016)

**DOI:** 10.1017/cts.2018.221

**Published:** 2018-11-21

**Authors:** Maria T. San Martin, Ruth Rios, Barbara Segarra, Karen G. Martinez, Estela Estape, Margarita Irizarry-Ramírez

**Affiliations:** University of Puerto Rico-Medical Sciences Campus

## Abstract

OBJECTIVES/SPECIFIC AIMS: The Hispanic Clinical and Translational Education and Career Development program entails formal research training (Phase I) through an established post-doctoral Master of Science in Clinical and Translational Research. The most qualified graduates from Phase I compete to receive 1–2 years support for continued mentoring and career development (Phase II program) aiming to apply for a regular research grant or career award (K or R series). OBJECTIVE: This project aims to present an evaluation of the Phase II program and Scholars outcomes. METHODS/STUDY POPULATION: METHODS: Participants (n=12) responded to a semistructured interview including 43 questions about program’s processes and outcomes. Descriptive and content analysis was done. RESULTS/ANTICIPATED RESULTS: RESULTS: Results show that 83% are women, 42% are MD, and 67% are affiliated to the University of Puerto Rico-Medical Sciences Campus and 67% were able to fulfill their career development expectations during the Phase II Award. At present (92%) are conducting clinical research in their current position. Outcomes include new selection of research line, K Awards, and enhanced skills in clinical and translational research DISCUSSION/SIGNIFICANCE OF IMPACT: DISCUSSION: Challenges identified were: time management, better coaching and a more structured mentoring experience. The main benefit of the program were protected time, research budget, and the opportunity to acquire more research experience.

